# Role of ryanodine receptor 2 and FK506-binding protein 12.6 dissociation in pulmonary hypertension

**DOI:** 10.1085/jgp.202213100

**Published:** 2023-01-10

**Authors:** Yong-Xiao Wang, Jorge Reyes-García, Annarita Di Mise, Yun-Min Zheng

**Affiliations:** 1 Department of Molecular and Cellular Physiology, Albany Medical College, Albany, NY, USA; 2 Departamento de Farmacología, Facultad de Medicina, Universidad Nacional Autónoma de México, Ciudad de México, México; 3 Department of Biosciences, Biotechnologies and Biopharmaceutics, University of Bari, Bari, Italy

## Abstract

Pulmonary hypertension (PH) is a devastating disease characterized by a progressive increase in pulmonary arterial pressure leading to right ventricular failure and death. A major cellular response in this disease is the contraction of smooth muscle cells (SMCs) of the pulmonary vasculature. Cell contraction is determined by the increase in intracellular Ca^2+^ concentration ([Ca^2+^]_i_), which is generated and regulated by various ion channels. Several studies by us and others have shown that ryanodine receptor 2 (RyR2), a Ca^2+^-releasing channel in the sarcoplasmic reticulum (SR), is an essential ion channel for the control of [Ca^2+^]_i_ in pulmonary artery SMCs (PASMCs), thereby mediating the sustained vasoconstriction seen in PH. FK506-binding protein 12.6 (FKBP12.6) strongly associates with RyR2 to stabilize its functional activity. FKBP12.6 can be dissociated from RyR2 by a hypoxic stimulus to increase channel function and Ca^2+^ release, leading to pulmonary vasoconstriction and PH. More specifically, dissociation of the RyR2–FKBP12.6 complex is a consequence of increased mitochondrial ROS generation mediated by the Rieske iron-sulfur protein (RISP) at the mitochondrial complex III after hypoxia. Overall, RyR2/FKBP12.6 dissociation and the corresponding signaling pathway may be an important factor in the development of PH. Novel drugs and biologics targeting RyR2, FKBP12.6, and related molecules may become unique effective therapeutics for PH.

## Teaser

ROS-dependent dissociation of FKBP12.6 mediates pulmonary artery vasoconstriction and pulmonary hypertension.

## Introduction

Pulmonary hypertension (PH) is a group of life-threatening lung diseases characterized by an increase in pulmonary arterial pressure ≥25 mmHg at rest or ≥30 mmHg during or after exercise (Prasad, 2019). According to clinical presentation, pathological findings, hemodynamic features, and treatment outcomes, the WHO divides PH into five groups: pulmonary arterial hypertension (PAH, group 1); PH due to left heart disease (group 2); PH due to chronic lung disease and/or hypoxia (group 3); PH due to pulmonary artery obstruction (group 4); and PH due to unclear multifactorial mechanisms (group 5; Mandras et al., 2020).

Regardless of differences in underlying pathogenic mechanisms, the pathophysiological features of the most common forms of PH include damage, i.e., plexiform lesions and vessel obliteration, and hyperproliferation of endothelial cells and smooth muscle cells (SMCs), vascular remodeling, and inflammation (Maarman et al., 2013). Inflammation consists of the deposition of macrophages, T cells, dendritic cells, mast cells, and B cells around the remodeled vessels. These cells contribute to the elevated serum levels of cytokines such as IL-1β, IL-6, IL-8, and CCL2 in PH patients (Price et al., 2012). Vascular remodeling promotes increased pulmonary vascular resistance, which increases right ventricular afterload and leads to hypertrophy and right ventricular failure (Singh et al., 2019; Maietta et al., 2021). Pulmonary vascular remodeling is extensively studied using animal models; the most commonly used models are hypoxia- and monocrotaline (MCT)-induced PH. These models exhibit important pathophysiological features of groups 1 and 3 of the WHO classification of PH (Maarman et al., 2013). Vascular cells from animals with chronic hypoxia-induced PH and from human patients with PAH, retain their dysregulated cell phenotypes such as pro-inflammation characterized by the synthesis and release of IL-6, CCL2, and VCAM-1, among others, and resistance to apoptosis in vitro (Hu et al., 2019). MCT-induced PH animals exhibit the same inflammatory markers, hemodynamics, and changes in the right ventricule and pulmonary vascular histology observed in patients with PAH (Maarman et al., 2013; Novelli et al., 2019). Therefore, in this review, the term pulmonary hypertension is used to refer to the subtypes PAH and hypoxia-related PH.

**Figure 1.**
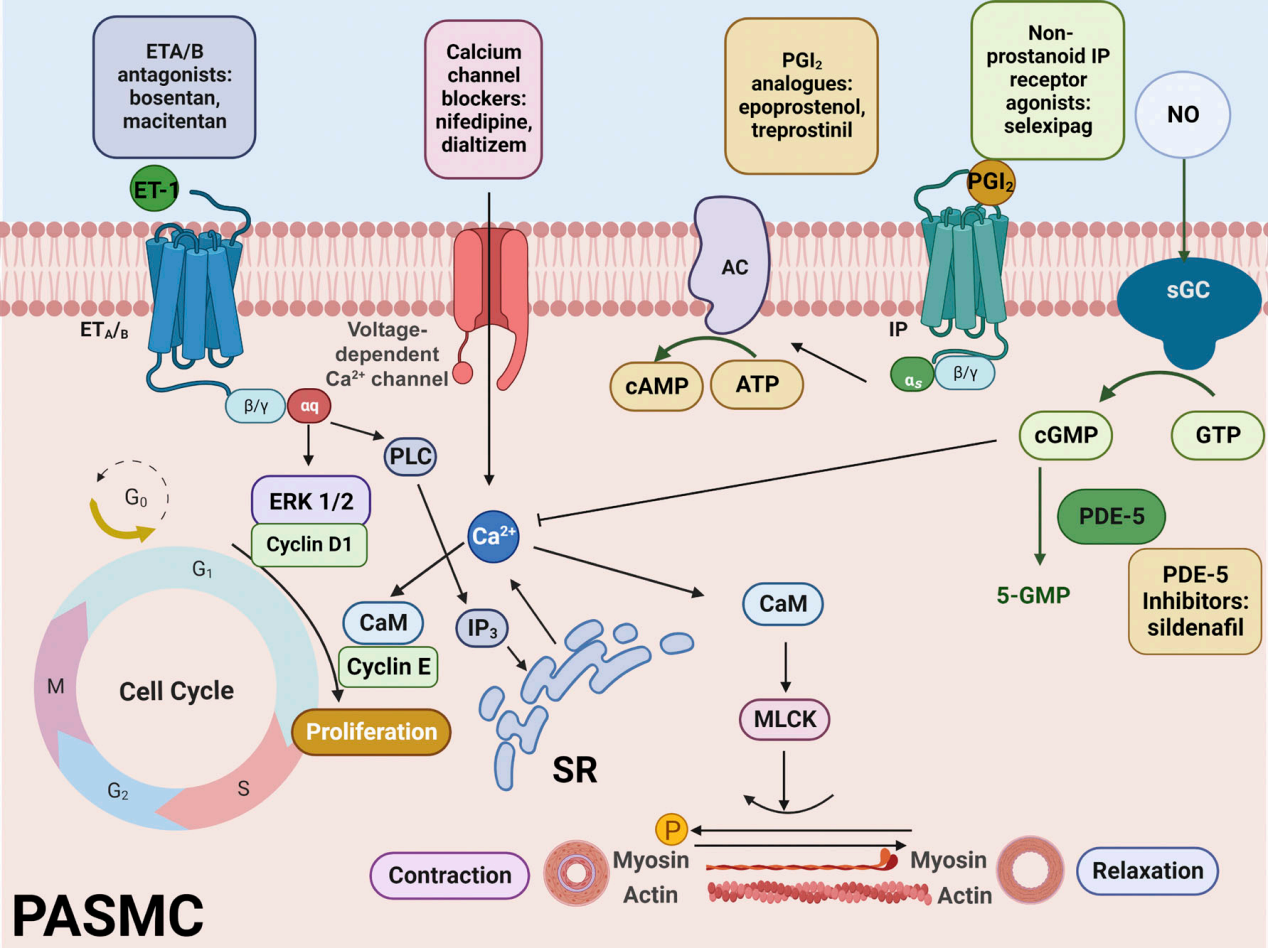
**Molecular mechanisms and drug targets for pulmonary hypertension.** The signaling pathways of endothelin-1 (ET-1), prostacyclin (PGI2), and nitric oxide (NO) are the three major targets for the treatment of PH. In PH, ET-1-mediated vasoconstriction occurs. ET-1 stimulates ET_A_ or ET_B_ receptors in the plasma membranes of PASMCs. These receptors are coupled to the phospholipase C (PLC) signaling cascade, which produces IP_3_ and triggers Ca^2+^ release from SR and voltage-dependent Ca^2+^ entry. The increase mediated by the mechanisms described above leads to activation of calmodulin (CaM), which in turn stimulates myosin light chain kinase (MLCK). This enzyme phosphorylates MLC, allowing actinomyosin movement and triggering contraction. There is also decreased production of PGI_2_ and NO. ET-1 signaling can be blocked with nonselective ET-1 receptor (ET_A_ or ET_B_) antagonists, including bosentan and macitentan. The PGI_2_ cascade can be activated by administration of PGI_2_ analogs or nonprostanoid PGI_2_ receptor (IP) agonists. This pathway stimulates the activity of adenylyl cyclase (AC), which triggers the formation of cyclic adenosine monophosphate (cAMP). The NO pathway can be enhanced by the use of PDE-5 inhibitors or by stimulation of soluble guanylate cyclase (sGC). In addition, endothelin-1 signaling may lead to activation of ERK1/2. This enzyme induces G_1_-GS transition and causes vascular smooth muscle proliferation Moreover, the Ca^2+^-CAM complex increases cyclin E activity and stimulates the G_1_-S transition, promoting vascular smooth muscle proliferation. Figure created with BioRender.com.

The increase in pulmonary arterial pressure is mediated by sustained contraction of pulmonary artery SMCs (PASMCs). This cellular response is triggered by an increase in intracellular Ca^2+^ concentration ([Ca^2+^]_i_). In SMCs, [Ca^2+^]_i_ is precisely regulated by several ion channels in the cell membrane, including voltage-dependent Ca^2+^ channels (VDCCs), transient receptor potential canonical channels (TRPCs), and store-operated Ca^2+^ channels (SOCCs). In addition, Ca^2+^-permeable channels in the sarcoplasmic reticulum (SR), including ryanodine receptors (RyRs), and inositol triphosphate receptors (IP_3_Rs) contribute to the regulation of [Ca^2+^]_i_ (Li et al., 2009; Song et al., 2015, 2017; Song et al., 2015; Song et al., 2017; Reyes-García et al., 2018; Li et al., 2021; Maietta et al., 2021; Reyes-García et al., 2021a; Reyes-García et al., 2021b). Furthermore, plasma membrane and sarcoplasmic Ca^2+^-ATPases maintain [Ca^2+^]_i_ at basal levels by extruding Ca^2+^ into the extracellular space or depositing it in the SR, thereby reducing free Ca^2+^ in the cytosol (Marín et al., 1999). The increase in [Ca^2+^]_i_ mediated by the channels described above leads to the activation of calmodulin (CaM), which in turn stimulates myosin light-chain kinase. This enzyme phosphorylates the myosin light chain, favoring actin to move across myosin and triggering contraction of SMCs (Fig. 1). In addition, Ca^2+^ increases, mainly through TRPCs (Wang et al., 2016; Qin et al., 2021), activate signal transduction pathways such as Ca^2+^-CAM, calcineurin/NFAT and mitogen-activated protein kinase (MAPK)-dependent pathways that lead to vascular smooth muscle (VSM) proliferation and remodeling (Fernandez et al., 2012). Increased synthesis of growth factors such as epidermal growth factor, endothelin-1, and angiotensin II is related to vascular injury in response to hypoxia and reactive oxygen species (ROS) production (Liu et al., 1995; Jernigan et al., 2004; Kim et al., 2015). The signaling pathway induced by these growth factors involves the increase of [Ca^2+^]_i_ and the activity of ERK1/2 and CAM. ERK1/2 may induce cyclins D1 and E leading to GS phase and proliferation (Zhang et al., 2003b; Karki et al., 2013). The Ca^2+^-CAM complex binds to cyclin E to initiate the transition from G_1_ to GS in VSM (Choi et al., 2006; Fig. 1).

The primary drug treatment for PH consists of the use of various types of vasodilators, including endothelin receptor antagonists, phosphodiesterase type 5 (PDE-5) inhibitors, inhaled nitric oxide, guanylate cyclase stimulators, prostacyclin (prostaglandin I_2_, PGI_2_) PGI_2_ analogs, non-prostanoid agonists of the PGI_2_ receptor, and Ca^2+^ channel blockers. These drugs target three major signaling pathways involved in abnormal pulmonary artery proliferation and contraction (Fig. 1). However, patients do not always respond well to these drugs (Yaghi et al., 2020; Tettey et al., 2021), opening the search for new therapeutic targets.

Hypoxia is an important factor in the development of PH. Chronic hypoxia promotes the remodeling of the pulmonary artery (PA) through the proliferation of SMCs and endothelial cells (Cahill et al., 2012). Moreover, hypoxia can increase the activity of RyR2 and induce the release of Ca^2+^ in PASMCs, leading to PA vasoconstriction, remodeling, and PH (Liao et al., 2011; Mei et al., 2020). Hypoxia-induced activation of RyR2 is attributable to the dissociation of FK506-binding protein 12.6 (FKBP12.6) from RyR2. In this context, hypoxia causes the production of ROS, primarily generated in the mitochondrial complex III and mediated by the Rieske iron-sulfur protein (RISP) in PASMCs (Rathore et al., 2008; Korde et al., 2011). RISP-mediated mitochondrial ROS may be responsible for hypoxia-triggered RyR2/FKBP12.6 dissociation and contribute to the development of PH (Liao et al., 2011). In this review article, we discuss the current information on the role of RyR2/FKBP12.6 dissociation and its regulation by hypoxia-induced ROS generation in the pathogenesis of PH.

## Physiology of RyRs in vascular smooth muscle

**Figure 2.**
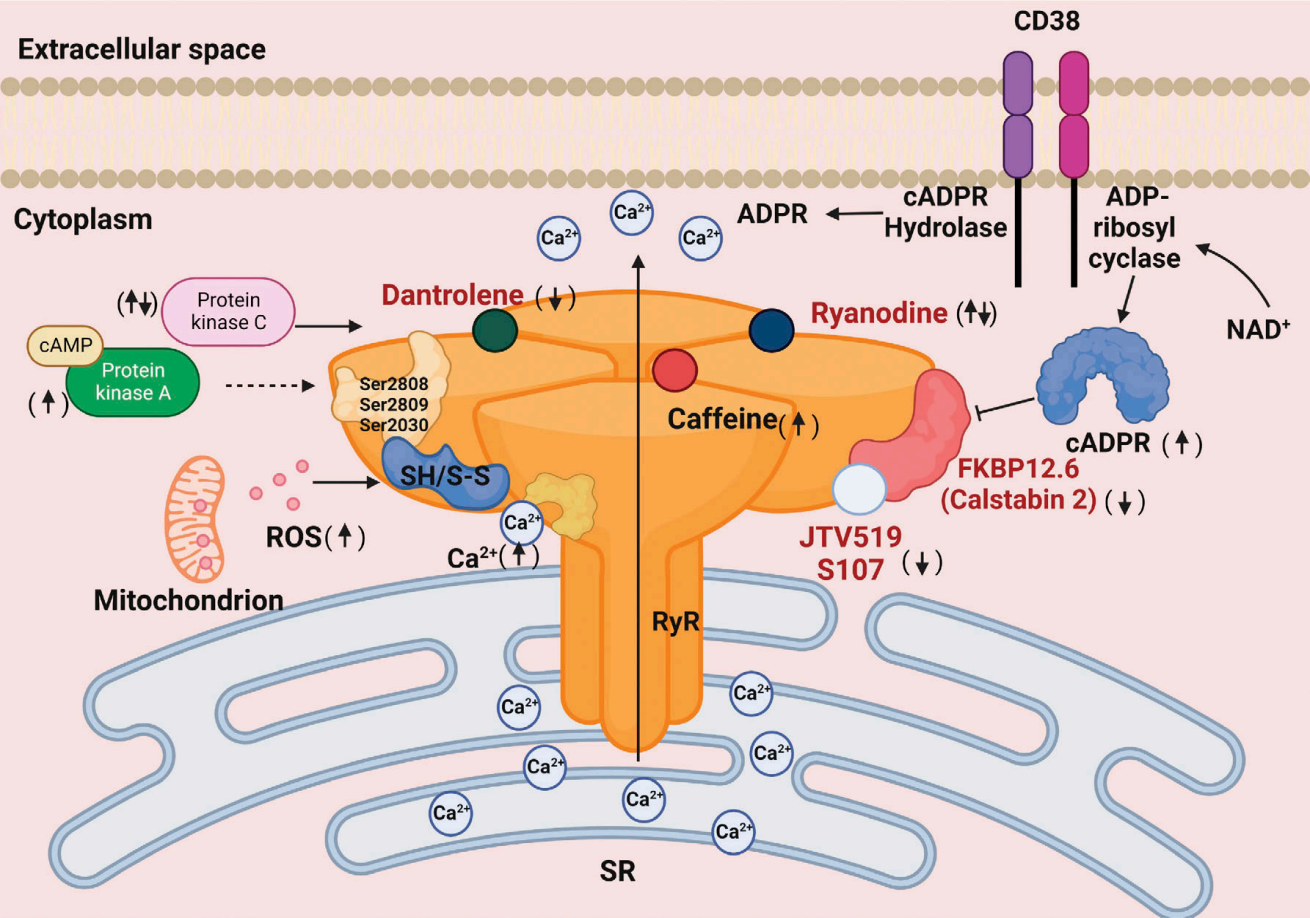
**Pharmacological and endogenous modulators of ryanodine receptor physiology.** The ryanodine receptor (RyR) is endogenously regulated by cADPR. cADPR is synthesized by CD38 from NAD^+^ and acts as a second messenger. CD38 is a key protein with ADP-ribosyl cyclase and cADPR hydrolase activity. Once cADPR is formed, it is rapidly hydrolyzed to inactive ADP-ribose under physiological conditions. FK506-binding protein 12.6 (FKBP 12.6) stabilizes RyR in its closed state. cADPR binds to FKBP12.6 and causes its dissociation from RyR, leading to channel activation. In cardiomyocytes, phosphorylation of RyR by protein kinase A (PKA) dissociates FKBP12.6 as well (dashed line). Phosphorylation by PKC can also modulate (promote or inhibit) the release of Ca^2+^ through the RyRs. ROS can oxidize (S-S) thiol groups present in RyR allowing its gating. Thiol groups can also be reduced (SH) by oxidative reactions. Ryanodine is a plant alkaloid used as an important pharmacological tool for characterizing the RyRs. Low concentrations of ryanodine cause long-lasting channel opening in the subconductance state, whereas high concentrations block the channel. Caffeine is a widely used pharmacological agonist of RyR. This methylxanthine increases the sensitivity of the RyR to cytosolic Ca^2+^ concentration and luminal Ca^2+^, allowing gating of the channel. Dantrolene is one of the best known RyR antagonists that keeps the channel in a closed state. JTV519 and S017 are benzothiazepine derivatives that stabilize RyR2 in its closed state by increasing the affinity of FKBP12.6 for this channel. The up and down arrows indicate whether the endogenous and pharmacological agents cause activation or inhibition of RyR. Figure created with BioRender.com.

RyRs are tetrameric proteins found in the peripheral membrane of the sarcolemmal SR junction of numerous cell types. In vertebrates, three RyR isoforms are molecularly characterized (RyR1, RyR2, and RyR3), all of which are found in VSM. These channels are responsible for the release of Ca^2+^ into the cytosol and trigger cellular functions such as contraction and proliferation (Kim et al., 2011; Pritchard et al., 2019). RyRs are mainly regulated by the binding of Ca^2+^ to the cytosolic side of the channel. The opening of RyRs is sensitive to [Ca^2+^]_i_ from 1 to 10 μM while millimolar [Ca^2+^]_i_ promotes their closed state (Dabertrand et al., 2013). RyRs are also endogenously regulated by cyclic ADP-ribose (cADPR), a derivative of nicotinamide adenine dinucleotide (NAD; Fig. 2). cADPR, as well as IP_3_ and nicotinic acid adenine dinucleotide phosphate (NAADP), are important messengers involved in intracellular Ca^2+^ release in VSMCs (Li et al., 2013; Wei et al., 2014). Synthesis of cADPR from NAD^+^ occurs by ADP-ribosyl cyclase activity and the major ADP-ribosyl cyclase in mammals is CD38, a transmembrane glycoprotein found in several tissues, including VSM (Evans and Dipp, 2002; Wei et al., 2014). CD38 can be activated by endogenous vasoconstrictors such as angiotensin II (Gul et al., 2008; Lee et al., 2015) and endothelin-1 (Giulumian et al., 2000; Thai and Arendshorst, 2008). The mechanism by which cADPR regulates the opening of RyRs is by the binding to FKBP12.6, leading to its dissociation from RyRs and the release of Ca^2+^ from the SR. In this context, Tang and colleagues demonstrated that the use of the anti-FKBP12 antibody blocks cADPR-induced activation of these channels (Tang et al., 2002). In addition, phosphorylation of RyRs by PKC modulates the release of Ca^2+^ through these channels (Peng et al., 2010). Several drugs modulate the Ca^2+^ sensitivity of RyRs or directly regulate the open/closed state of these channels (Zheng et al., 2005; Zheng et al., 2008). For instance, low concentrations (i.e., 0.1 μM) of the alkaloid ryanodine exert agonistic effects and promote local/spontaneous events of Ca^2+^ release named Ca^2+^ sparks, whereas higher concentrations have antagonistic effects on RyRs. Furthermore, the methylxanthine caffeine activates all RyR isoforms at concentrations >5 mM (Essin and Gollasch, 2009).

RyRs are involved in global and localized Ca^2+^ increases (Li et al., 2009; Li et al., 2021). In VSM cells (VSMCs) and other SMCs, these receptors can be activated by Ca^2+^ influx through plasma membrane Ca^2+^ channels or by Ca^2+^ release from neighboring IP_3_Rs or other RyRs. This mechanism is known as Ca^2+^-induced Ca^2+^ release (CICR), which plays an essential role controlling vascular tone and excitation–contraction coupling (Kotlikoff, 2003; Ureña et al., 2007; Liu et al., 2009; Kaßmann et al., 2019). Ca^2+^ sparks are associated with vasodilation. Ca^2+^ sparks trigger K^+^ efflux through Ca^2+^-activated K^+^ channels with high conductance (BK). K^+^ efflux leads to spontaneous transient outward currents (STOCs) that result in cell membrane hyperpolarization and disrupt Ca^2+^ entry through L-type VDCC (L-VDCC; Zhao et al., 2017; Kaßmann et al., 2019). The importance and involvement of RyRs in global and localized Ca^2+^ increments depend on the RyR isoform. We have shown in PASMCs that RyR1 plays a critical role in the CIRC process following high K^+^-triggered membrane depolarization (which induces the opening of VDCCs; Li et al., 2009; Li et al., 2021). RyR2 appears to be the major contributor to global and spontaneous Ca^2+^ release in systemic and pulmonary arteries. In this context, Kaßmann and colleagues showed that caffeine-induced contraction of the aorta, cerebral, and mesenteric arteries was abolished when smooth muscle RyR2 conditional knockout (KO) were used (Kaßmann et al., 2019). The same authors also demonstrated that caffeine was unable to trigger contraction of the SM-RyR2 KO lung. Furthermore, RyR2 seems the major SR Ca^2+^ release channel involved in the generation of Ca^2+^ sparks in VSMCs. Genetic deletion of RyR2 abrogates Ca^2+^ sparks and STOCS in freshly isolated tibial and mesenteric artery VSMCs (Kaßmann et al., 2019). These results contrast with the findings of Coussin and colleagues that both RyR1 and RyR2 contribute to the generation of Ca^2+^ sparks in cultured VSMCs from the portal vein (Coussin et al., 2000). These results could be due to specific tissue differences or to the fact that cultured VSMCs may not represent the physiology of native VSMCs because protein expression changes during cell dedifferentiation. With respect to RyR3, Löhn and colleagues found that Ca^2+^ sparks and STOCs are enhanced in cerebral VSMCs from RyR3^−/−^ mice compared with those from wild-type mice and suggested that this isoform is responsible for regulating Ca^2+^ sparks generated by RyR1 and RyR2. Moreover, they showed that RyR3 did not appear to be involved in global Ca^2+^ responses as caffeine in RyR3 KO VSMCs elicited normal global Ca^2+^ increases, while the Ca^2+^ spark cycle can be determined only by the other two isoforms, RyR1 and/or RyR2 (Löhn et al., 2001). The different Ca^2+^ functions of RyRs may be due to their localization. For example, in PASMCs, RyR1 is localized in the periphery and perinuclear SR regions, whereas RyR2 is localized in the SR periphery near the sarcolemmal membrane and RyR3 is expressed in perinuclear regions (Yang et al., 2020).

## Hypoxia and pulmonary vasoconstriction

Hypoxia-induced pulmonary vasoconstriction (HPV) has been known for years (Voelkel, 1986). HPV aids to redirect blood flow from hypoxic to better ventilated regions of the lung as a protective response. Nevertheless, a serious decrease in alveolar oxygen can lead to vascular damage and remodeling, persistent vasoconstriction, and PH (Post et al., 1995). The hypoxic response of isolated pulmonary vessels is biphasic (Evans and Dipp, 2002). Phase I represents the early transient vasoconstriction that peaks at 5 min, whereas phase II involves the slowly progressive and tonic vasoconstriction that reaches plateau at 30–60 min (Bennie et al., 1991; Aaronson et al., 2002; Weissmann et al., 2004; Weissmann et al., 2006b). The sustained vasoconstriction can last over a period of 2–8 h (Balanos et al., 2003; Cheng et al., 2017). Multiple membranal ion channels leading to increased [Ca^2+^]_i_, and phosphorylation processes are involved in both phases of HPV (Waypa and Schumacker, 2006; Weir and Olschewski, 2006; Wang et al., 2007; Waypa et al., 2013). Phase I is triggered by the closure of voltage-gated K^+^ channels in the plasma membrane of hypoxia-sensitive PASMCs, leading to depolarization and opening of VDCCs, and contraction of VSM (Post et al., 1992; Archer et al., 1993; Post et al., 1995; Evans et al., 1998; Osipenko et al., 1998). Acute hypoxia and subsequent HPV increase mean pulmonary artery pressure, leading to overperfused areas of the lung, stress failure of pulmonary capillaries, and edema formation (Young et al., 2019). It is also proposed that vasoconstriction favors shear stress that in turn triggers the proliferation of VSMCs (Voelkel and Tuder, 2000). All of these factors contribute to the development of PH.

**Figure 3.**
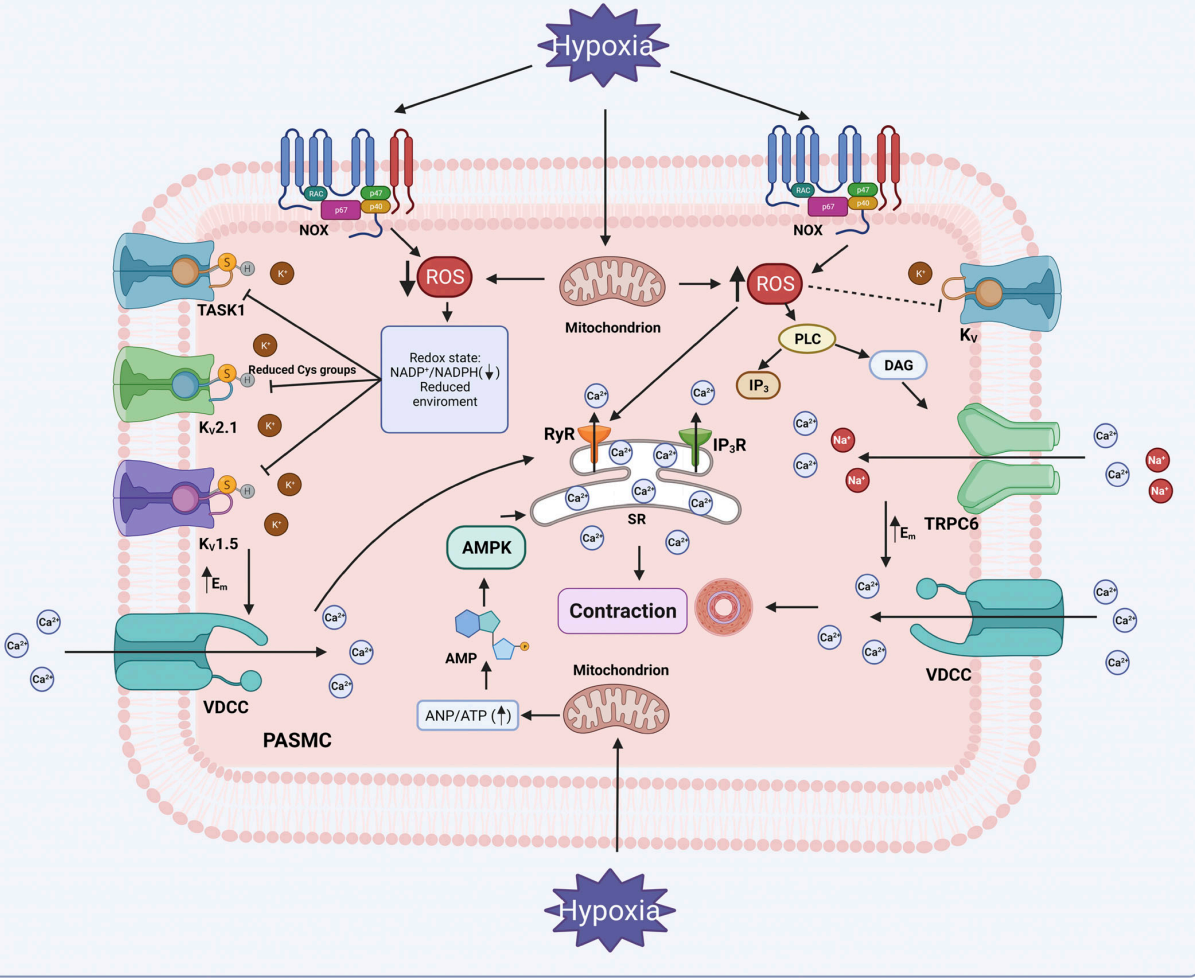
**Cellular mechanisms of hypoxic pulmonary vasoconstriction.** Three main mechanisms, dependent on ROS production and redox status, have been proposed to explain the cellular basis of pulmonary vasoconstriction. The first theory postulates that hypoxia leads to decreased production of ROS by NADPH oxidase (NOX) and mitochondria, resulting in a reduced environment. The decreased NADP^+^/NADPH ratio leads to a reduction of cysteine groups (Cys) in K^+^ channels, probably K_V_1.5, K_V_2.1, and TASK-1. This change favors the closure of K^+^ channels and the increase in membrane potential (Em, depolarization), as well as the opening of voltage-dependent Ca^2+^ channels (VDCC). Ca^2+^ influx promotes the release of Ca^2+^ from the SR, which triggers myosin and actin activity and vasoconstriction. The second hypothesis states that hypoxia leads to increased formation of ROS. ROS stimulates the action of phospholipase C (PLC) and the associated production of inositol triphosphate (IP_3_) and diacylglycerol (DAG). IP_3_ promotes Ca^2+^ release from IP_3_ receptors (IP_3_R) in SR, and DAG activates transient receptor potential canonical channel 6 (TRPC6). This channel allows Ca^2+^ and Na^+^ influx and promotes depolarization and opening of VDCCs. In addition, ROS trigger the opening of RyRs, allowing the release of more Ca^2+^ from SR. Furthermore, the increased production of ROS may inhibit K_V_ channels (dashed line), contributing to membrane depolarization. Finally, the third hypothesis states that hypoxia promotes a shift in the energy production cycle, leading to increased production of adenosine monophosphate (AMP), which stimulates AMP-dependent kinase (AMPK), increasing intracellular Ca^2+^ through SR. Figure created with BioRender.com.

Several ROS and redox balance-related mechanisms are proposed to explain O_2_ sensing and contraction in PASMCs. Nonetheless, controversy exists about the source of ROS, their target mechanisms, and whether they increase or decrease in response to hypoxia (Michelakis et al., 1995; Waypa and Schumacker, 2006). One of the first hypotheses (the Redox hypothesis) states that oxidative phosphorylation in mitochondria and ROS production decrease after hypoxia, promoting the cytosol of PASMCs enter to a more reduced state and triggering the inhibition of redox-sensitive K^+^ channels, including K_V_1.5 and K_V_2.1 (Michelakis et al., 1995; Archer et al., 1998; Mehta et al., 2008). This theory is supported by the fact that the reducing agent DTT decreases K^+^ currents and causes membrane depolarization, while the oxidizing agent DTNB increases K^+^ currents and promotes hyperpolarization in PASMCs (Fig. 3; Olschewski et al., 2004). Moreover, O_2_ is the main substrate for the generation of ROS, so it is conceivable that the production of ROS decreases under hypoxic conditions. However, an increase in mitochondrial ROS formation is also reported in hypoxic environment in PASMCs, as postulated by the ROS hypothesis (Leach et al., 2001; Liu et al., 2003; Waypa et al., 2013). This hypothesis states that even in the presence of O_2_ depletion, mitochondrial ROS production (mainly H_2_O_2_) is increased, triggering RyRs-mediated Ca^2+^ release and the inhibition of K_V_ channels (Post et al., 1995; Cogolludo et al., 2006; Mei et al., 2020). A third hypothesis, the Energy hypothesis, describes the shift in energy production from oxidative phosphorylation to glycolysis caused by hypoxia-induced mitochondrial dysfunction and reduced production of ROS. This alteration leads to improved production of AMP, which activates AMPK and increases [Ca^2+^]_i_ through the Ca^2+^ release from SR (Freund-Michel et al., 2014; Evans et al., 2015).

Phase II of HPV depends on Ca^2+^ sensitization of smooth muscle myofilaments mediated by PKC and RhoA/Rho kinase (ROCK). ROCK phosphorylates the myosin-binding subunit of MLC phosphatase (MLCP), MYPT-1, and the MLCP inhibitor protein CP-17. Both phosphorylations inhibit the activity of MLCP and enhance contraction of SMCs (Robertson et al., 1995; Jernigan et al., 2008). Chronic hypoxia improves the activity and expression of Rho and ROCK (Broughton et al., 2008; Wang et al., 2019). This signaling pathway stimulates the expression of hypoxia-inducible factor (HIF)-1α, which in turn upregulates the expression and function of TRPC1 and TRPC6 channels in PASMCs. Thus, increased [Ca^2+^]_i_ and pulmonary artery contraction via these channels are improved after hypoxia (Wang et al., 2019). TRPC6 is also involved in acute hypoxic pulmonary vasoconstriction through hypoxia-induced accumulation of diacylglycerol (DAG) and consequent activation of this TRPC isoform (Weissmann et al., 2006a). Membrane-localized Ca^2+^ increases can lead to the opening of BK channels; however, these K^+^ channels do not appear to be involved in HPV because their blockade does not cause depolarization of PASMCs or increase normoxic pulmonary vascular resistance (Archer and Michelakis, 2002). Chronic hypoxia and the attendant phase II of the HPV, and the activity of HIF-1α promote the switch of PASMCs from the contractile to the synthetic phenotype that underlies the proliferation and remodeling of the pulmonary vasculature (Michelakis et al., 2002; Mam et al., 2010; Fernandez et al., 2012; Dunham-Snary et al., 2017). For example, persistent vasoconstriction elicits the expression of TGF-β, PDGF, and ICAM-1 molecules that contain shear stress response elements, thus mediating vascular proliferation and remodeling during chronic hypoxia, and HIF-1α induces the transcription of VEGF (Voelkel and Tuder, 2000; Deudero et al., 2008).

Moreover, other membrane channels are linked to HPV and PH. For example, K_V_3.1b possesses oxygen-sensing properties and is blocked by hypoxia (Osipenko et al., 2000). In addition, the expression of the α-subunit of the K_V_7.4 channel is downregulated in hypoxic pulmonary vasculature. Additionally, oral administration of the nonspecific K_V_7 channels activator (flupirtine, 30 mg/kg/d) for 5 d prevented hypoxia-evoked increased vascular resistance (Sedivy et al., 2015). Another type of K^+^ channel recently described in PASMCs, TWIK-related acid-sensitive K^+^ channel 1 (TASK-1), is associated with regulation of resting membrane potential and vascular tone because of its voltage independence (Gurney et al., 2003). TASK-1 is sensitive to hypoxia (Olschewski et al., 2006), and the expression of this channel is reduced in PH patients (Antigny et al., 2016). Cl^−^ channels activity is also associated with HPV and PH. The activity and the expression of the Ca^2+^-activated Cl^−^ channel, ANO1/TMEM16A, are augmented by chronic hypoxia in PASMCs from rats and PH patients (Sun et al., 2012; Papp et al., 2019). Increased activity of these channels may enhance vasoconstrictor agonist-induced membrane depolarization and subsequent opening of VDCCs, further increasing vasoreactivity after hypoxia. Nevertheless, using a model of isolated perfused/ventilated mouse lung, Jain et al. (2020) demonstrated that TMEM16A or other Ca^2+^-activated Cl^−^ channels are not involved in pulmonary vasoconstriction induced by alveolar hypoxia. Finally, RyR2 is susceptible to ROS-mediated oxidation (following hypoxia) of thiol groups present in the channel (Maietta et al., 2021). This modification enhances RyR2 activity and increases [Ca^2+^]_i_, which promotes HPV (Truong et al., 2020).

## RyR2 plays an important role in PH

Despite the presence of the three subtypes of RyRs (RyR1, RyR2, and RyR3) in PASMCs, each appears to have distinct functional roles with the RyR2 subtype being the major player in hypoxic responses (Truong et al., 2020). We have shown using smooth muscle-specific RyR2 KO, RISP knockdown, or FKBP12.6 KO mice that RyR2 contributes substantially to hypoxia-induced vasocontraction and pulmonary artery remodeling (Zheng et al., 2008; Liao et al., 2011; Mei et al., 2020). Moreover, RyR2 is the subtype responsible for mediating CICR in cardiac and airway smooth muscle (Liu et al., 2009; Benitah et al., 2021). In light of this, we have proposed that hypoxia may cause RyR2 channel opening, leading to Ca^2+^ release from SR in PASMCs and contributing to increased and sustained vasoconstriction, which plays an important role in the development of PH (Mei et al., 2020).

Considering the above hypothesis, we induced acute hypoxic reactions in pulmonary artery tissues. These tissues were stimulated with normoxic and hypoxic physiological saline for 5 min. After hypoxia exposure, maximal ryanodine binding to RyRs was significantly increased in PAs compared with controls (normoxic stimulated). Moreover, 5 min of hypoxia exposure caused a strong increase in [Ca^2+^]_i_ in freshly isolated PASMCs, which was blocked in cells from RyR2 knockout mice. These in vitro assays demonstrate that acute hypoxia increases Ca^2+^ release through RyR2 in the pulmonary artery (Liao et al., 2011). Furthermore, using a chronic hypoxia-induced PH murine model and ryanodine-binding assays, we found that maximal ryanodine binding was greatly augmented in PASMCs from hypoxic mice compared with cells from normoxic mice and the dissociation constant of ryanodine binding was decreased (Mei et al., 2020). We also showed that caffeine administration triggered an intracellular Ca^2+^ increase in PASMCs from hypoxic mice that was markedly heightened compared with cells from normoxic mice. In the same work, we showed that the caffeine-induced intracellular Ca^2+^ increase, which was enhanced by hypoxia, was completely inhibited in cells from RyR2 KO mice, ruling out the contribution of RyR1 and RyR3 (Mei et al., 2020). Both approaches suggest that RyR2-associated Ca^2+^ release is significantly increased during acute or chronic hypoxia.

As mentioned earlier, the persistent vasoconstriction and remodeling of the pulmonary artery are the main pathophysiological features of PH (Maietta et al., 2021). We reported that the administration of norepinephrine elicited much greater pulmonary vasoconstriction in hypoxic mice compared with normoxic mice. Increased wall thickness was observed as well in middle and large pulmonary arteries from mice exposed to chronic hypoxia (Mei et al., 2020). We also found that enhanced hypoxia-induced pulmonary vasoconstriction was abolished in RyR2 knockout mice. The chronic hypoxia triggered increase in wall thickness in middle and large pulmonary arteries is also blocked in RyR2 knockout mice (Mei et al., 2020). Furthermore, we did not observe any change in RyR2 expression under hypoxia.

Hypoxia and RyR2-associated PA remodeling are mediated by the nuclear factor κ B (NF-κB)/cyclinD1 pathway (Mei et al., 2020). NF-κB is a transcription factor involved in triggering inflammation and cell proliferation (Truong et al., 2021). NF-κB is strongly activated in PA endothelial and SMCs from PAH patients compared with healthy controls (Price et al., 2013). In addition, pathogenic gene variants of two signaling molecules that regulate NF-κB, namely TNF interacting protein 2, and TNF receptor associated factor 2 are implicated in the development of PAH (Pienkos et al., 2021), and the inhibition of the NF-κB signaling cascade has a therapeutic effect on PH (Hosokawa et al., 2013; Li et al., 2014). In the resting state, the NF-κB subunit p50/p65 complex binds to NF-κB inhibitor alpha (IκBα), which sequesters this dimer in the cytoplasm. Inflammatory stimuli can cause the degradation of IκBα to allow the p50/p65 complex to enter the nucleus and trigger a transcriptional process (Truong et al., 2021). Chronic hypoxia increases NF-κB levels in the nuclear extract of lungs from exposed rats 13-fold compared with control animals (Sarada et al., 2008). Moreover, Patel and colleagues observed that hypoxia increased NF-κB activity in mouse lungs and cultured endothelial cells, leading to increased expression of endothelin-1 and the ICAM1 (Patel et al., 2017), key mediators involved in proliferation and remodeling of vascular cells (Tian et al., 2020). Regarding the role of RyR2 in hypoxia-induced PA remodeling, Mei and colleagues observed that chronic hypoxia causes increased expression of the p65/p50 complex in the nuclei of PASMCs, and that this enhancement is attenuated in RyR2 knockout mice. They also observed that the expression of IκBα is decreased in PASMCs from CH mice, consistent with the fact that degradation of IκBα is required for NF-κB translocation. NF-κB can regulate the promoter of cyclin D1 (an essential mediator in the cell cycle) to induce proliferation of SMCs and remodeling of PA (Zeng et al., 2010; Raghavan et al., 2012). Consistent with this, Mei and colleagues also demonstrated that cyclin D1 expression is upregulated in PASMCs from mice treated with chronic hypoxia but not in RyR2 knockouts. In vivo administration of the NF-κB inhibitor pyrrolidine dithiocarbamate (PDTC) suppresses the upregulation of cyclin D1 in PASMCs from mice with PH and prevents PA remodeling and blocks the increased right ventricular systolic pressure in mice exposed to chronic hypoxia (Mei et al., 2020).

Right ventricular systolic pressure and hypertrophy are two of the most reliable indicators of functional status and prognosis in PH (Ryan and Archer, 2014). In this context, we found that knockout of RyR2 abrogated the hypoxia-induced increase in right ventricular systolic pressure. Moreover, the increase in right ventricular weight is also completely blocked by RyR2 knockout (Mei et al., 2020). These and the above findings indicate that RyR2 knockout mice do not develop PH and open the possibility to further investigate this channel as a therapeutic target. We also demonstrated that in vivo treatment with the well-known RyR antagonist, tetracaine, abolishes the hypoxia-induced increase in right ventricular weight and systolic pressure (Mei et al., 2020).

## The role of RyR2 in PH is attributed to its dissociation from FKBP12.6

FK506-binding proteins (FKBPs) are peptidyl-prolyl isomerases that serve as intracellular targets for the immunosuppressant FK506 (Tacrolimus) and rapamycin. FKBPs are endogenous regulators of RyR2 function studied in detail in cardiac tissue. Two members of the FKBP family are expressed in the mammalian heart: FKBP12 (also known as calstabin1) and FKBP12.6 (also known as calstabin2). In cardiac tissue, the complex formed by FKBP12.6 and the RyR2 subtype is highly implicated in the regulation of Ca^2+^ signaling and excitation–contraction coupling. FKBP12.6 maintains the RyR2 channel in its closed state, resulting in reduced activity (Xin et al., 2002; Gonano and Jones, 2017). Furthermore, removal of FKBP12.6 from the RyR2 channel by FK506 or rapamycin increases the opening probability of the RyR2 channel and leads to a sub-conductance state (Gonano and Jones, 2017; Maietta et al., 2021). Cardiac hypertrophy is an essential marker for PH (Frey et al., 2004; Lunde et al., 2011; Shimizu and Minamino, 2016; von Siebenthal et al., 2016). In this context, Xiao and colleagues found that the left ventricular mass in FKBP12.6 deficient mice was significantly augmented compared with wild-type hearts (41.0 vs. 34.1%, respectively) after the infusion of angiotensin II. Moreover, they demonstrated that adequate function of FKBP12.6 protects the heart from angiotensin-induced cardiac hypertrophy by inhibiting Ca^2+^/CaM-mediated signaling cascades including calcineurin/NFAT and AKT/mTOR pathways (Xiao et al., 2018).

**Figure 4.**
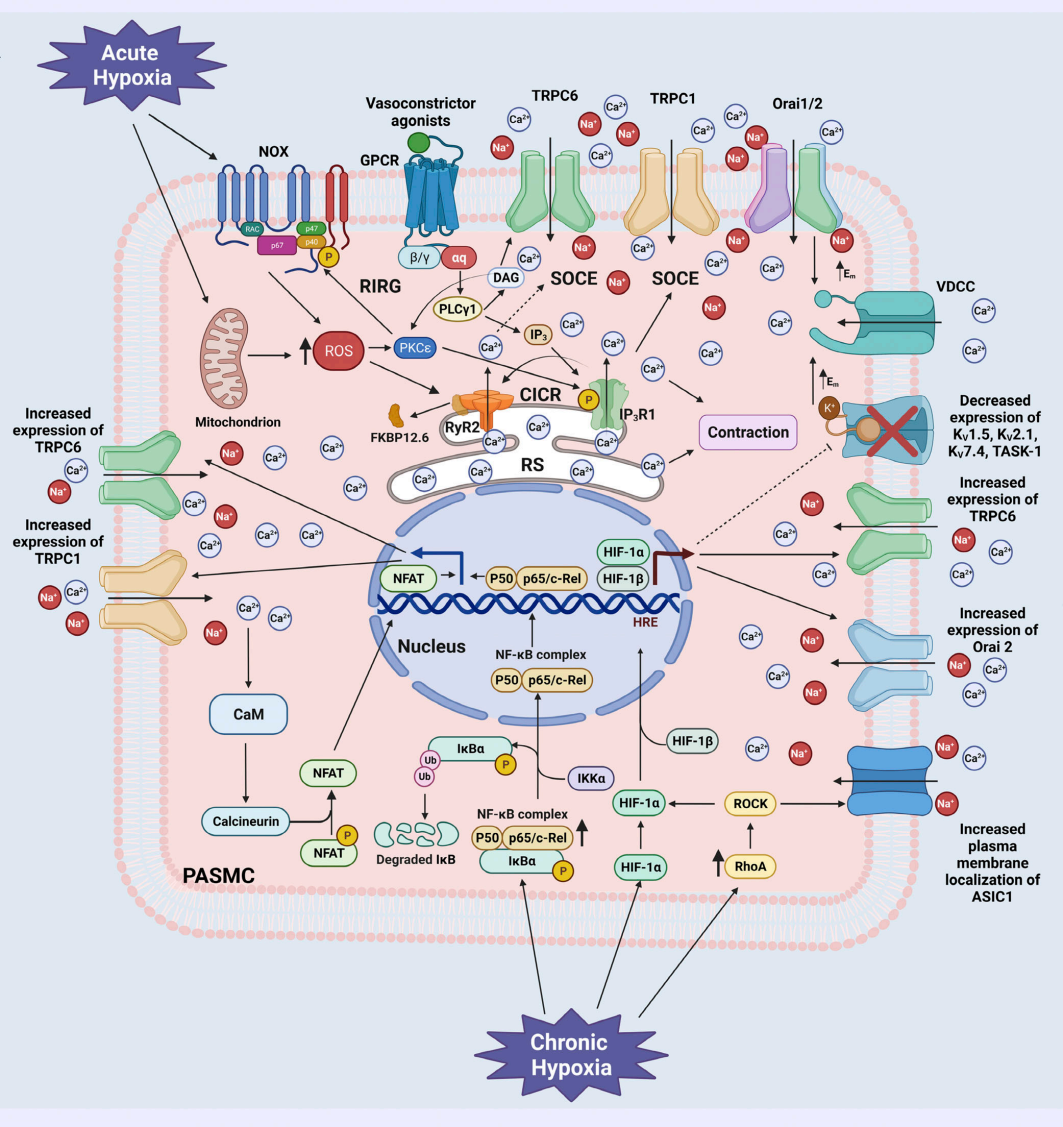
**Schematic representation of the effects of acute and chronic hypoxia on dissociation of FKBP 12.6 from RyR2 in PASMCs.** Acute hypoxia triggers the formation of NOX and mitochondrial ROS. The increased cytosolic ROS may activate protein kinase C-ε (PKCε). PKCε stimulates NOX to initiate further ROS-generation. This ROS-induced ROS-generation (RIRG), together with hypoxia-induced direct mitochondrial ROS-generation, leads to synergistic disassociation of FKBP12.6 from RyR2, increasing channel activity and inducing Ca^2+^ release from the SR. Mitochondrial ROS and vasoconstrictor agonists through activation of a G protein-coupled receptor (GPCR) stimulate phospholipase C-γ (PLCγ) signaling. PLCγ induces the formation of inositol triphosphate (IP_3_) and diacylglycerol (DAG), which causes the opening of IP_3_R1 and the release of Ca^2+^ from the SR. DAG can activate TRPC6, triggering the influx of Ca^2+^ and Na^+^. In addition, depletion of SR elicits store-operated Ca^2+^ entry (SOCE) mediated by store-operated Ca^2+^ channels (SOCCs) such as TRPC6, TRPC1, and Orai1/2. Ca^2+^ released through IP_3_R1 induces Ca^2+^ release through RyR2 (CIRC). Furthermore, depletion of SR induced by activation of RyR2 can trigger SOCE (dashed line) independently of the IP_3_ pathway. Chronic hypoxia stimulates hypoxia-inducible factor-1α (HIF-1α) to enter the nucleus and activate hypoxia response elements (HRE) to induce TRPC6 and Orai2 expression. Chronic hypoxia also increases the expression and activity of RhoA, which stimulates Rho-associated protein kinase (ROCK). ROCK triggers translocation of HIF-1α to the nucleus and membrane localization of ASIC1. Similarly, chronic hypoxia enhances the expression of NF-κB, and the signaling pathway of this transcription factor induces the expression of TRPC1 and TRPC6. Sustained Ca^2+^ entry through the upregulated channels activates the calmodulin/calcineurin/NFAT pathway, which also increases the expression of TRPC1. In addition, the expression of K_V_1.2, K_V_2.1, K_V_7.4, and TASK-1 is downregulated, probably by the action of HIF-1α (dashed line). Na^+^ influx across SOCCs and decreased expression of K^+^ channels lead to membrane depolarization and opening of VDCCs. Finally, Ca^2+^ overload promotes sustained pulmonary arterial vasoconstriction, remodeling, and hypertension. Additional abbreviations: P, phosphorylation; IκBα, NFκ-B inhibitor α; IKK α, inhibitor-κB kinase α; ASIC1, acid-sensitive ion channel 1; NFAT, nuclear factor of activated T cells; VDCCs, voltage-dependent Ca^2+^ channels; CaM, calmodulin. Figure created with BioRender.com.

As for the pulmonary artery, we have reported that hypoxia causes dissociation of FKBP12.6 from RyR2, which increases the activity of this channel and allows the release of Ca^2+^ from the SR (Fig. 4; Zheng et al., 2004; Liao et al., 2011; Mei et al., 2020; Truong et al., 2020; Yang et al., 2020). In this context, in 2004, our research group demonstrated the presence of FKBP12 and FKBP12.6 in equine and murine PASMCs. However, the same work showed that FKBP12.6 interacts with only RyR2 and not with any other subtype, i.e., RyR1 or RyR3 (Zheng et al., 2004). In the same work, we observed that a hypoxic stimulus increased Ca^2+^ release through RyR2 in PASMCs from FKBP12.6-deficient mice compared with wild-type mice. Later, we found that hypoxia promoted the dissociation of FKBP12.6 from RyR2 by causing its translocation to the cytoplasmic space (Liao et al., 2011). Moreover, in PASM tissues from hypoxic mice and from patients with PH, Mei et al. (2013) reported that the ratio of RyR2/FKBP12.6 was significantly decreased. The same authors also found that proliferation of PASMCs was greatly increased in FKBP12.6-deficient mice (Mei et al., 2020). These mice developed increased sensitivity to chronic hypoxia-induced PH, as they exhibited higher right ventricular systolic pressure compared with wild-type mice under normoxic conditions. These FKBP12.6-associated effects require further investigation because the deletion of FKBP12.6 suggests a PH phenotype that should not improve after hypoxia. However, we did not observe a phenotype of FKBP12.6-deficient mice or any effect of FK506 treatment under normoxic conditions (Mei et al., 2020). Apparently, removal of FKBP12.6 alone cannot lead to PH. Accordingly, both FKBP12.6 and RyR2 contribute to the development of hypoxic PH.

The orally active benzothiazepine derivative S107 blocks intracellular Ca^2+^ release by stabilizing the RyR1/FKBP12 complex in skeletal muscle (Mei et al., 2013). Furthermore, S107 stabilizes the RyR2/FKBP12.6 association in PASMCs, preventing chronic hypoxia-induced PH (Mei et al., 2020). Specifically, the treatment with S107 diminishes the augmented ratio of SR Ca^2+^ leak from SR Ca^2+^ stores in PASMCs from mice exposed to chronic hypoxia and completely inhibits hypoxia-induced pulmonary vasoconstriction in vivo. Moreover, this benzothiazepine derivative also abolishes hypoxia-induced pulmonary vascular remodeling and prevents the hypoxia-induced increase in right ventricular systolic pressure. Furthermore, oxidation of RyR2 in cardiac myocytes triggers Ca^2+^ leak from the SR and contributes to right ventricular dysfunction and heart failure PH (Huang et al., 2021). Therefore, pharmacological stabilization of the RyR2/FKBP12.6 complex in the pulmonary artery could serve to prevent or treat PH. However, because other organs such as the heart may also be affected, further research and development of a pharmacological delivery system specifically for the pulmonary artery are needed.

## Implications of RyR2/FKBP12.6 on IP_3_Rs and store operated Ca^2+^ entry

Because RyRs are Ca^2+^-sensitive proteins, it is conceivable that Ca^2+^ efflux through IP_3_Rs may lead to the opening of RyRs that trigger an even greater SR release of Ca^2+^ followed by vasoconstrictor agonists (Li et al., 2009; Li et al., 2021). This crosstalk between RyRs and IP_3_Rs may be influenced by the dissociation of FKBP12.6 from the RyRs (Zheng et al., 2004; Maietta et al., 2021). IP_3_Rs are the functional units responsible for Ca^2+^ increase and further vasoconstriction after stimulation of G protein coupled receptors (GPCRs; Zhang et al., 2003a; Zheng et al., 2004; Zheng et al., 2008; Li et al., 2009; Liao et al., 2011; Li et al., 2021). Ca^2+^ release by the phospholipase C (PLC)-IP_3_ signaling cascade recruits and activates neighboring domains of RyRs, resulting in a massive increase in cytosolic Ca^2+^ (Gordienko and Bolton, 2002; Zhang et al., 2003a). FKBP12.6 regulates the increase in [Ca^2+^]_i_ triggered by norepinephrine and the associated contraction of the pulmonary artery (Zheng et al., 2004). Stimulation of FKBP12.6-deficient PASMCs with norepinephrine triggers a much greater increase in [Ca^2+^]_i_ than in control cells. Accordingly, norepinephrine evokes a stronger vasocontraction response in pulmonary arteries of FKBP12.6-deficient mice (Zheng et al., 2004). Moreover, the crosstalk between RyRs and IP_3_Rs through CICR and the concomitant depletion of IP_3_Rs-regulated Ca^2+^ stores activate store operated Ca^2+^ entry (SOCE) in PASMCs (Zhang et al., 2003a). However, Lin and colleagues observed in PASMCs that SR-Ca^2+^ release through RyRs (using the RyR agonist 4-CmC) induced a membranal Ca^2+^ influx that was independent of the IP_3_Rs-mediated SOCE signaling pathway, suggesting a role for RyRs (particularly RyR2) in triggering the activity of SOCCs in these cells (Lin et al., 2016). In this context, SR Ca^2+^ depletion is detected by the Ca^2+^ sensor stromal interaction molecule 1 (STIM1) and STIM2, which oligomerize and translocate to the junction between SR membrane and plasmatic membrane to associate with SOCCs, such as Orai1, 2, and 3, as well as TRPC1, 4, and 6, and ASIC1 channels (Jernigan et al., 2009; Fernandez et al., 2012; Trebak, 2012; Fernandez et al., 2015; Lin et al., 2016; Wang et al., 2017). RyRs-gated Ca^2+^ entry or SOCE mediated by RyRs, requires depletion of Ca^2+^ stores and a specific conformational change (Lin et al., 2016). Lin and colleagues observed that caffeine, unlike 4-CmC, was unable to activate SOCE, probably due to different binding sites (Fessenden et al., 2003; Fessenden et al., 2006) of the two RyR agonists. In addition, mutation of the binding site of 4-CmC, I4827, in the C-terminus of RyRs abolishes RyR-gated SOCE. In support, caffeine and 4-CmC are known to induce different conformational changes in RyRs (Liu et al., 2010; Lin et al., 2016). According to Lin and colleagues, these requirements suggest a physical or functional association of RyR with the molecular components of SOCE in PASMCs (Lin et al., 2016). Accordingly, Sampieri and colleagues have demonstrated the functional coupling between RyR1 and TRPC1 in CHO cells (Sampieri et al., 2005). In addition, in HEK cells, RyR1 coimmunoprecipitates with TRPC3 (Kiselyov et al., 2000), and in the same cells RyR2 colocalizes with STIM1 (Thakur et al., 2012).

It is well-known that hypoxia elicit the release of intracellular Ca^2+^ stores and activate SOCE in PASMCs (Ng et al., 2005; Ng et al., 2008; Peng et al., 2013). With this respect, chronic hypoxia upregulates the expression of TRPC6 and Orai2 by mediating the activity of HIF-1α, increasing SOCE and [Ca^2+^]_i_ in PASMCs at rest (Wang et al., 2006; Wang et al., 2017). Moreover, chronic hypoxia can trigger the activity of NFAT, leading to augmented expression of TRPC1 (Wang et al., 2009). Mei and colleagues also found that chronic hypoxia increased the expression of TRPC1 and TRPC6 and diminished the expression of K_V_ channels, likely through an NF-κB dependent mechanism (Mei et al., 2020). Regarding K^+^ channels, they found that release of Ca^2+^ by RyR2 resulted in decreased expression of K_V_1.5 after chronic hypoxia treatment and that knockdown of RyR2 restored its expression (Mei et al., 2020). Furthermore, using a proximity ligand assay, Herbert and colleagues showed that RhoA and ASIC1 colocalized in PASMCs, and hypoxia stimulated the activity of the former to promote ASIC1 plasma membrane localization and Ca^2+^ entry (Herbert et al., 2018). Collectively, acute and chronic hypoxia play important roles in triggering SOCE, which is likely mediated by RyR2 in PASMCs, i.e., acute hypoxia increases ROS that stimulates RyR2 opening and depletion of SR. This process triggers the interaction of STIM1 and Orai1 to further promote the interaction with Orai2, TRPC1, and TRPC6, leading to SOCE (Reyes et al., 2018). Moreover, TRPCs and Orai2 are upregulated by chronic hypoxia in PASMCs, such that RyR2 serves as a primary molecule in the hypoxic Ca^2+^ signaling alternations in PASMCs.

## Dissociation of RyR2/FKBP12.6 is primarily mediated by mitochondrial ROS

It is generally accepted that changes in intracellular ROS concentration ([ROS]_i_), due to mitochondrial dysfunction in pulmonary artery endothelial cells and PASMCs, contribute to the development of PH (Maietta et al., 2021). ROS are highly reactive chemicals formed as a by-product of the normal metabolism of O_2_ (Tejero et al., 2019; Veith et al., 2019). These noxious byproducts may serve as signaling molecules. For instance, ROS can oxidize multiple Ca^2+^ channels, including the RyR2, to promote intracellular Ca^2+^ increases (Liao et al., 2011; Oda et al., 2015; Kobayashi et al., 2021). This process is known as ROS-induced Ca^2+^ release (RICR). In addition, intracellular Ca^2+^ can modulate the production of ROS in a process called Ca^2+^-induced ROS generation (CIRG; Feno et al., 2019; Truong et al., 2021). Two main sources of ROS are known: the electron transport chain (ETC) in mitochondria and NAPDH oxidase (NOX) in the cell membrane and cytosol (Tejero et al., 2019; Veith et al., 2019). Hypoxia can lead to an increase in the production of ROS (Korde et al., 2011; Smith and Schumacker, 2019). In this context, Marshall and colleagues described the presence of NOX in PASM and showed that hypoxia leads to the production of superoxide in this tissue. They were the first group to propose NOX as an O_2_-sensing mechanism to trigger HPV (Marshall et al., 1996). In addition, Waypa and colleagues demonstrated that mitochondria in PASMCs also serve as O_2_ sensors during hypoxia and that complex 3 in these organelles generates ROS in response (Waypa et al., 2001).

The release of Ca^2+^ from the SR through RyR2 plays an important role in the hypoxic increase of [Ca^2+^]_i_ in PASMCs, leading to vasoconstriction and PH (Mei et al., 2020). In PASMCs, hypoxia-induced production of ROS can stimulate RyR2 (Korde et al., 2011; Liao et al., 2011; Truong et al., 2020). Using the ROS detection probe dichlorodihydrofluorescein/diacetate (H2DCF/DA), we demonstrated that acute hypoxic stimulation significantly increased the production of ROS in PASMCs (Wang et al., 2007; Korde et al., 2011; Liao et al., 2011). Furthermore, using a specific biosensor (HyPer, for tracking intracellular hydrogen peroxide) to determine ROS production, we also detected hypoxia-induced increase in ROS generation in isolated mitochondria of PASMCs (Korde et al., 2011). We as well confirmed that the increase in ROS was generated in mitochondria after exposure to hypoxia using MitoTracker to stain mitochondria and DCF to determine ROS generation. Following hypoxia, the generation of ROS is significantly increased in both mitochondrial and non-mitochondrial regions. However, the production of ROS is triggered earlier and more strongly in mitochondrial regions than in non-mitochondrial regions (Wang et al., 2007). Production of ROS through the ETC involves the action of the enzymatic complexes I (NADH: ubiquinone oxidoreductase), II (succinate dehydrogenase), and III (cytochrome bc1 complex; Maietta et al., 2021). Using specific inhibitors of these complexes, including rotenone (complex I), nitropropionic acid (complex II), and myxothiazol (complex III), our research group found that the ETC complex I and II and III actively produced ROS (particularly H_2_O_2_) in response to hypoxia in PASMCs, even though complex III seems to be more important. ROS produced in this complex are responsible for the dissociation of FKB12.6 from RyR2 (Rathore et al., 2006; Korde et al., 2011; Yadav et al., 2013).

## NOX is an important resource for hypoxic ROS generation in PASMCS, but it is secondary to mitochondrial ROS

NOX corresponds to a family of enzyme complexes located in the cell membrane or outer mitochondrial membrane (Veith et al., 2019). NOXs catalyze the transfer of electrons to O_2_ to form O_2_^−^ and H_2_O_2_, two types of ROS. NOX family includes seven members, NOX 1–5 and DUOX 1–2. NOX1, NOX2, NOX4, and NOX5 are expressed in the constitutive cells of the blood vessel wall (VSMCs, endothelial cells, and fibroblasts; Rivera et al., 2010). However, in PASMCs, NOX4 appears to contribute mostly to the formation of ROS (Ward, 2008). The active form of these enzyme complexes comprises six subunits, including the membrane-bound subunits p22^phox^ and gp91^phox^ and the cytosolic subunits p47^phox^ and p67^phox^ (Rathore et al., 2008; Tejero et al., 2019; Maietta et al., 2021). Several works have described that inhibition of NOXs can abolish vasoconstriction induced by hypoxia (Zhang et al., 1997; Weissmann et al., 2000; Liu et al., 2006; Weissmann et al., 2006b).

We have reported the presence of NOX1 and NOX4, but not NOX2 in mouse PASMCs (Rathore et al., 2008). In these cells, acute hypoxia exposure leads to a sharp increase in the NOX activity and an increase in the translocation of p47^phox^, a key component in the formation of active NOX to the plasma membrane, leading to the formation of ROS. In addition, we also demonstrated that genetic deletion of the NOX p47^phox^ subunit in PASMCs reduced hypoxic ROS formation and hypoxic increase in [Ca^2+^]_i_ (Rathore et al., 2008).

Hypoxia can trigger protein kinase C-ε (PKCε) activity (Rathore et al., 2006; Rathore et al., 2008). This PKC isoform is associated with myocardial protection against hypoxic damage (Gray et al., 2004) and is involved in initiating hypoxic vasoconstriction (Littler et al., 2003). In this context, we have shown that inhibition of PKC blocks hypoxia-induced activation of NOX (Rathore et al., 2006; Rathore et al., 2008). Moreover, the hypoxia-induced increase in PKCε activity is completely blocked by the mitochondrial inhibitors rotenone and myxothiazol. These findings suggest that the activation of NOX, which is triggered by hypoxia in PASMCs, is mediated by the mitochondrial ROS-PKCε signaling axis (Rathore et al., 2006). This process in which the activity of PKCε (induced by ROS) stimulates NOX to further increase ROS formation and [ROS]_i_, is termed ROS-induced ROS generation (RIRG) in PASMCs (Rathore et al., 2006; Wang et al., 2007; Rathore et al., 2008). More importantly, regulation of NOX activity by mitochondrial ROS and PKCε is implicated in the development of hypoxia-induced pulmonary vasoconstriction (Wang et al., 2007) and may contribute to the differential responsiveness to hypoxia in the pulmonary artery and other vascular tissues. Furthermore, in PASMCs from rats, the mechanism of Ca^2+^ release induced by Ang II is mediated in part by activation of CD38 through NOX2-dependent ROS production, resulting in synergistic Ca^2+^ release from Ca^2+^-gated cADPR stores (Lee et al., 2015).

## RISP is the primary molecule for mitochondrial ROS generation in PASMCs

Among all ETC complexes, the complex III, also known as ubiquinol-cytochrome *c* oxidoreductase, is singled out for its great ability to generate ROS (Turrens et al., 1985; Rana et al., 2000; Waypa and Schumacker, 2006; Powers et al., 2011; Saldana-Caboverde et al., 2020). The complex III contains a catalytic subunit called RISP, which is involved in electron transfer and ATP synthesis (Saldana-Caboverde et al., 2020; Truong et al., 2021). RISP is also implicated in the formation of ROS after hypoxia (Guzy et al., 2005; Korde et al., 2011; Truong et al., 2020).

In 2005, Guzy and colleagues demonstrated that the complex III, and in particular RISP, is required for mitochondrial ROS production in Hep3B, HEK293, and 143B cells (Guzy et al., 2005). They showed that RISP triggers the stabilization of HIF-1α and the formation of ROS after hypoxia. Subsequently, ROS diffuse into the cytosol where they serve as oxygen sensors in conjunction with mitochondria. Accordingly, Korde and colleagues proved that RISP is a primary molecule for hypoxia-induced mitochondrial ROS production in PASMCs (Korde et al., 2011). They performed experiments in which they silenced or overexpressed RISP in these cells. These experiments showed that transfection of control small-interference RNAs (siRNAs) had no effect on baseline ROS production in PASMCs. However, the RISP siRNAs decreased the baseline production of ROS, and the silencing of RISP in PASMCs almost completely blocked hypoxia-induced ROS generation in the isolated complex III and the hypoxia-induced ROS formation in isolated mitochondria (Korde et al., 2011). In contrast, overexpression of RISP increased hypoxia-ROS generation in isolated complex III and mitochondria from PASMCs (Korde et al., 2011).

RISP is essential for triggering Ca^2+^ rise and vasoconstriction in response to hypoxia in PASMCs (Korde et al., 2011; Mei et al., 2020; Truong et al., 2020). ROS generated in the mitochondrial complex III can oxidize the RyR2 and trigger its hyperactivity, i.e., increased sensitivity and Ca^2+^ release (Andersson et al., 2011; Liao et al., 2011; Dridi et al., 2020). Using a DNP antibody-based protein oxidation assay, Mei and colleagues found considerable RyR2 oxidation in PASMCs from hypoxic mice compared with cells from normoxic mice. However, knocking down RISP in vivo blocked RyR2 oxidation (Mei et al., 2020). Moreover, the increase in [Ca^2+^]_i_ and hypoxia-induced vasoconstriction after RyR2 oxidation were largely blocked by silencing RISP in PASMCs and pulmonary artery, respectively (Korde et al., 2011; Mei et al., 2020). Mei and colleagues also found that the expression of FKBP12.6 was reduced in SR from PASMCs of hypoxic mice, but not the expression of RyR2 (Mei et al., 2020). All these results suggest that hypoxia acts through two main mechanisms: on the one hand, oxidation of RyR2 is induced, which facilitates dissociation of FKBP12.6, and on the other hand, the amount of FKBP12.6 that could be bound to RyR2 is reduced, which increases the activity of RyR2 and promotes sustained vasoconstriction in PH (Mei et al., 2020). Moreover, knockdown of RISP in vivo blocks the hypoxia-triggered increase in right ventricular pressure and abolishes the hypoxia-triggered increase in right ventricular weight (Mei et al., 2020).

In addition to the effects of hypoxia on RyR2, Yadav and colleagues reported in 2013 that acute hypoxia can also regulate IP_3_Rs by increasing PLCγ1 activity in PASMCs (Yadav et al., 2013). Later, the same authors demonstrated that mitochondrial ROS formation after hypoxia or exogenous ROS (500 μM) also increases PLCγ1 activity by promoting its phosphorylation at tyrosine-783. Activated PLCγ1 leads to the formation of IP_3_ (Yadav et al., 2018), which stimulates the IP_3_R1 isoform and releases Ca^2+^ in the cytosol, causing hypoxic vasoconstriction. It is reported that mitochondrial ROS production after hypoxia triggers IP_3_R1 phosphorylation by PKCε, which increases IP_3_ binding and triggers a large increase in [Ca^2+^]_i_ (Rathore et al., 2006; Rathore et al., 2008; Yadav et al., 2018). Accordingly, mice exposed to chronic hypoxia show increased PLCγ1 activity and enhanced pulmonary artery vasoconstriction (Yadav et al., 2018).

RISP is also involved in the hypoxic PLCγ1-IP_3_ signaling pathway in PASMCs. Specific suppression of RISP expression with lentiviral short hairpin RNAs (shRNAs) prevents mitochondrial ROS formation and inhibits hypoxia-triggered increased PLCγ1 activity (Yadav et al., 2018). Moreover, pharmacological inhibition of the complex III with myxothiazol also abrogates the hypoxia-induced increase in PLCγ1 activity in PASMCs (Korde et al., 2011; Yadav et al., 2018). Conceivable, RISP is a master regulator of the hypoxic increase in PLCγ1 action, followed by mitochondrial ROS production in the complex III. More importantly, RISP regulates the dissociation of FKBP12.6 from RyR2, which may amplify the Ca^2+^ increase produced by the augmented activity of IP_3_R1, leading to improved and persistent pulmonary artery vasoconstriction and likely increased vascular resistance, which contributes to vascular remodeling and the development of PH (Liang et al., 2022).

## RyR2-mediated Ca^2+^ release causes RISP-dependent mitochondrial ROS production to further enhance the hypoxia-induced ROS generation and cellular responses

ROS generated in mitochondria can lead to further formation of ROS through activation of NOX and eventually to an even greater increase in [ROS]_i_ (Rathore et al., 2008; Maietta et al., 2021). The process described above appears to be a feed-forward system that enhances ROS and Ca^2+^ responses and promotes improved vasoconstriction seen in PH. The increased [Ca^2+^]_i_ promoted in hypoxic or PH PASMCs appears to be poorly regulated by plasma membrane Ca^2+^-ATPase because its expression is suppressed by PDGF, an essential mediator in vascular remodeling during PAH development (Deng et al., 2021). Additionally, the activity of SR Ca^2+^-ATPase and ATP binding are disrupted by ROS (Sharov et al., 2006; Cook et al., 2012). A balancing effect may be exerted by the BK channels. Although these channels do not appear to be involved in HPV like K_V_ channels, long-term hypoxia increases the Ca^2+^ affinity of BK channels and thus their activity. This positive regulation could serve as an acclimation response to regulate vascular tone in response to hypoxia (Tao et al., 2015). Despite this finding, further research is needed to elucidate the mechanisms that counteract the enhanced ROS and Ca^2+^ responses proposed in this review.

However, Ca^2+^ released into the cytosol through SR channels can also be taken up by mitochondria. Mitochondria-dependent Ca^2+^ regulation is involved in several functions, including mitochondrial fusion and fission, ROS generation, redox signaling, autophagy, and cell death. As concerns for PASMCs, Drummond and Tuft demonstrated that caffeine and ATP trigger an increase in both [Ca^2+^]_i_ and mitochondrial [Ca^2+^] ([Ca^2+^]_m_; Drummond and Tuft, 1999). [Ca^2+^]_m_ was detected with the fluorescent indicator rhod-2, whereas [Ca^2+^]_i_ was measured at the same time with fura-2. The authors showed that [Ca^2+^]_m_ continued to increase when [Ca^2+^]_i_ peaked and remained elevated even when [Ca^2+^]_i_ began to decrease. In addition, the uncoupler of mitochondrial oxidative phosphorylation, FCCP, prolonged the time to recovery of basal [Ca^2+^]_i_ after caffeine exposure (Drummond and Tuft, 1999), indicating the involvement of mitochondria in the buffering and removal of cytosolic Ca^2+^ in these cells. Gurney and colleagues confirmed Ca^2+^ signaling between SR and mitochondria in arterial SMCs, with the latter organelle playing an important role in returning [Ca^2+^]_i_ to basal levels after SR activation by vasoconstrictor agonists (Gurney et al., 2000). Mitochondrial Ca^2+^ uptake needs that this organelle be located close within a microdomain of high [Ca^2+^] as occurs near to receptors in SR (McCarron et al., 2013). Moreover, mitochondrial Ca^2+^ uptake may improve or diminish the amplitude of Ca^2+^ signals (McCarron et al., 2013). For instance, mitochondria in SMCs are located near IP_3_Rs clusters to regulate IP_3_-mediated Ca^2+^ release (Chalmers and McCarron, 2009). Additionally, mitochondria modulate Ca^2+^ transients through RyRs, i.e., inhibition of mitochondrial function with FCCP or cyanide prolongs the cytoplasmic Ca^2+^ transient evoked by caffeine in aortic SMCs (Gurney et al., 2000). Furthermore, mitochondrial function regulates subplasmalemmal Ca^2+^ dynamics in VSMCs. With respect to VDCCs, mitochondrial Ca^2+^ uptake produces an accelerated decrease of the Ca^2+^ transient across these channels; however, the rate of increase of the Ca^2+^ transient does not appear to be altered by this organelle function (McGeown et al., 1996; Drummond and Tuft, 1999). Mitochondrial uncoupling also abolishes Ca^2+^ sparks (Cheranov and Jaggar, 2004) and spontaneous transient inward currents (STICS, Ca^2+^ activated Cl^−^ currents; Greenwood et al., 1997), and reduces STOCS (Ca^2+^ activated K^+^ currents; Cheranov and Jaggar, 2004). Overall, it mitochondria dynamically modulate Ca^2+^ signaling in the range of 200 nM to 10 μM and have difficulty modulating high local [Ca^2+^] levels near activated VDCCs (McCarron et al., 2012; McCarron et al., 2013).

Ca^2+^ and ROS signaling and dynamics between mitochondria and the SR in PASMCs were recently studied by Yang et al. (2020). They demonstrated that caffeine and norepinephrine (Li et al., 2021) increased Ca^2+^ (due to the release of Ca^2+^ by RyRs and IP_3_Rs, respectively) and subsequently [ROS]_i_ in PASMCs. Moreover, increased mitochondrial [ROS] ([ROS]_m_) was observed in isolated mitochondria after PASMCs were exposed to caffeine or norepinephrine. Using mit-2mutAEQ, the mitochondria-targeted double mutant aequorin Ca^2+^ sensor, Yang and colleagues also found that norepinephrine, caffeine, and hypoxia can increase [Ca^2+^]_m_ and this increase was blocked by the mitochondrial Ca^2+^ uniporter (MCU) inhibitor Ru360 (10 μM) in the pulmonary artery (Yang et al., 2020). MCU is one of the major proteins involved in Ca^2+^ uptake. This transporter is responsible for controlling Ca^2+^ movement through the microdomain of mitochondrial and SR membranes (Song et al., 2017). Downregulation of MCU and the resulting imbalance of [Ca^2+^]_m_ and [Ca^2+^]_i_ induces cell proliferation and migration and promotes the development of PH (Hong et al., 2017).

Yang and colleagues also found that exogenous Ca^2+^ (3–200 μM) enhanced the formation of ROS in mitochondria and the complex III, which were isolated from PASMCs, showing that CIRG occurs in these cells (Yang et al., 2020). Moreover, they demonstrated that this process is caused only by the activity of the complex III and not by others. In this context, the formation of ROS triggered by caffeine is attenuated in RISP-deficient isolated mitochondria and PASMCs. Moreover, Ru360 abrogates caffeine-triggered ROS formation in PASMCs, strongly suggesting that Ca^2+^ released by RyR2 is responsible for mitochondrial ROS formation (Yang et al., 2020). Accordingly, Ru360 attenuates exogenous Ca^2+^-induced ROS formation in isolated mitochondria (Yang et al., 2020). Since hypoxia is able to increase the activity of RyR2 and the formation of ROS and promote the development of PH (Wang et al., 2007; Liao et al., 2011; Mei et al., 2020), Yang and colleagues also investigated the role of MCU in hypoxia-induced formation of ROS. The authors discovered that pharmacological blockade of MCU abrogates hypoxia-triggered ROS formation in PASMCs and in isolated mitochondria from Ru360-exposed PASMCs (Yang et al., 2020). Moreover, pharmacological inhibition and genetic downregulation of RyR2 attenuate hypoxic ROS formation and the increase in [ROS]_m_. Altogether, RyR2-mediated Ca^2+^ release following the hypoxic mitochondrial ROS formation triggers the activity of MCU and subsequently the increase of RISP-dependent [ROS]_m_ in PASMCs. It is conceivable that this versatile signaling pathway plays an important role in hypoxic vasoconstriction and in the development and progression of PH.

## Therapeutic potential and clinical relevance of targeting RyR2/FKBP12.6 pathway

The RyR2/FKBP12.6 complex is proposed to be a target for some cardiac diseases. Moreover, RyR2 dysfunction is associated with heart failure (HF) and atrial fibrillation (AF; Alvarado and Valdivia, 2020; Zhang et al., 2021). Multiple missense mutations in RyR2 are related to arrhythmogenic right ventricular cardiomyopathy type 2 (Tiso et al., 2001), and catecholaminergic polymorphic ventricular tachycardia (CPVT; Priori et al., 2001; Duan et al., 2018), two inherited forms of sudden cardiac death. RyR2 mutations occur clustered in the N-terminal domain, the central domain, and the channel-forming domain. In this context, Oda and colleagues showed that a defective interaction between the N-terminal and central domains occurs in hearts with cardiac arrest (Oda et al., 2005). This abnormality caused the interacting N-terminal and central domains corresponding to the Gly^2460^-Pro^2495^ region of RyR2 to become detached from each other (unzipped), which facilitates the dissociation of FKBP12.6 from the channel and leakage of Ca^2+^ (Yamamoto et al., 2008). Therefore, several drugs have been evaluated for their ability to inhibit RyR2 or its accessory proteins in the heart. A large number of chemical compounds such as propafenone, tetracaine, hydantoin, and their derivatives which normalize RyR2 activity have been developed. However, only benzothiazepine derivatives (K201 and S107) can block the interaction between RyR2 and FKBP12.6 (Connell et al., 2020).

K201, also called JTV519, was developed to provide a stronger protective effect against Ca^2+^-induced myocardial damage (Kaneko, 1994; Kaneko et al., 2009). This drug is a benzothiazepine derivative studied in phase II trials for the treatment of myocardial infarction (James, 2007) and AF (Connell et al., 2020). Although K201 has multiple functions, such as blocking Na^+^, K^+^, and Ca^2+^ channels (Kimura et al., 1999; Kiriyama et al., 2000; Nakaya et al., 2000; Hasumi et al., 2007; Kaneko et al., 2009) and blocking the α_1_-adrenoreceptor (Kaneko, 1994; Kaneko et al., 2009), this drug stabilizes RyR2 in its closed state by increasing the affinity of FKBP12.6 for this channel (Wehrens et al., 2004). In this way, Ca^2+^ leakage is prevented, conferring protection against contractile dysfunction and ventricular arrhythmias (Toischer et al., 2010; Otani et al., 2013). In addition, the action of K201 attenuates the progression of HF due to Ca^2+^ overload and the resulting damage to the myocardium (Dincer, 2012). S107, the derivative of K201, has not yet been assessed in clinical trials. However, in a mouse model of CPVT, K201 inhibits RyR2 Ca^2+^ leak and prevents cardiac arrhythmias (Lehnart et al., 2008). As well, Guo and colleagues demonstrated that S107 blocked increased basal Ca^2+^ release and improved cardiac performance in a model of RNA-binding protein 20 (RBM20) cardiomyopathy (Guo et al., 2021). Finally, administration of S107 (10 μM) in isoproterenol-stimulated cardiomyocytes from a CPVT patient reduced pro-arrhythmic delayed after depolarizations (DADs) to 25% (Sasaki et al., 2016).

Regarding PH and pulmonary vessels as a target, no treatments based on the interaction of RyR2/FKBP12.6 or other proteins associated with this channel have been developed or tested in humans. However, because right ventricular failure is a major cause of death in patients with PH, stabilization of RyR2 in the heart is postulated as a treatment option to increase survival. A study conducted by Huang and colleagues showed that administration of the RyR2 stabilizer dantrolene to decompensated right ventricular cardiomyocytes reduced the frequency of Ca^2+^ sparks. Moreover, intraperitoneal administration of this drug attenuated the progression of right ventricular failure and prolonged the survival of 23% of rats with PH induced by MCT (Huang et al., 2021). Furthermore, dantrolene inhibited the dissociation of calmodulin from RyR2, preventing Ca^2+^ sparks in hypertrophied right ventricular cardiomyocytes (Tanaka et al., 2022). In the same work, chronic dantrolene treatment prevented right ventricular expansion and suppressed collagen levels in an animal model of MTC-induced PH. In addition, this RyR2 stabilizer prevented ventricular tachycardia induced by the combination of caffeine and epinephrine. All these dantrolene effects increased animal survival by 80% (Tanaka et al., 2022). We demonstrated that in vivo treatment of mice exposed to chronic hypoxia with S107 attenuated the increased RyR2 activity of PASMCs, i.e., S107 inhibited the chronic hypoxia-induced dissociation of FKBP12.6 from RyR2 and attenuated increased Ca^2+^ leak. S107 also abolished PA remodeling by hampering chronic hypoxia-induced muscularization and SMC proliferation and eliminated right ventricular hypertrophy (Mei et al., 2020). In this review, we propose that stabilization of RyR2 by inhibiting dissociation of FKBP12.6 may be an effective therapeutic agent against PH. However, more specific inhibitors and appropriate routes of administration should be developed to avoid cardiac side effects. Further basic and clinical research is needed on this topic.

## Conclusion

Exacerbated PASMC contraction and remodeling are common markers of PH. These processes appear to be mediated to a large extent by an enhanced increase in [Ca^2+^]_i_. Several channels are involved in Ca^2+^ processing in SMCs. This review article highlights the importance of RyR2 in the control of Ca^2+^ homeostasis and ROS generation mediated by hypoxia, as well as its role in the development of PH. We and other investigators have well documented that hypoxia is highly specifically involved in the generation of ROS and increases in [Ca^2+^]_i_ in PASMCs, serving as of the main cause of PA vasoconstriction, remodeling and PH. Accordingly, a complex signaling pathway involving the hypoxia-induced mitochondrial ROS generation and then RyR2-dependent Ca^2+^ release, termed RICR process, is well described in this article.

Nevertheless, acute and chronic hypoxia both can increase RISP-dependent mitochondrial ROS generation in PASMCs. The increased mitochondrial ROS can enter the cytosol and activate PKCε. The activated PKCε stimulates NOX to trigger further ROS generation. This ROS-induced ROS, together with the hypoxia-induced direct mitochondrial ROS production, synergistically dissociate FKBP12.6 from RyR2, which increases channel activity, and then induces the release of Ca^2+^ from the SR. The released Ca^2+^ causes PA vasoconstriction, PA remodeling, and eventually PH. Moreover, RyR2-mediated Ca^2+^ release also causes RISP-dependent mitochondrial ROS production, which further enhances hypoxia-induced ROS generation and cellular responses. The signaling that links the SR to mitochondria through a CIRG process represents an important pathway in hypoxia-induced PH. It is conceivable that pharmacological and genetic stabilization/inhibition of the RyR2/FKB12.6 complex and RyR2 per se in SMCs could be the novel and effective therapeutic options in the treatment of PH.
